# A rare case report of ileosigmoid knots that cause intestinal obstruction during Ramadan fasting

**DOI:** 10.1016/j.ijscr.2024.110725

**Published:** 2024-12-09

**Authors:** Hanene Zenati, Mohamed Ali Chaouch, Mohamed Zayeti, Ramzi Beltaifa, Besma Gafsi, Faouzi Noomen

**Affiliations:** a Department of Visceral and Digestive Surgery, Monastir University Hospital, Monastir, Tunisia; b Department of Anesthesia, Monastir University Hospital, Monastir, Tunisia

**Keywords:** Ileosigmoid knotting, Case report, Intestinal obstruction

## Abstract

**Introduction and importance:**

This case report aims to highlight the clinical presentation, diagnostic challenges, surgical intervention, and subsequent management strategies of ISK during Ramadan fasting.

**Case presentation:**

52-Year-old male with a three-day history of symptoms of intestinal obstruction. He complained of abdominal distention, vomiting, and absolute constipation. He had hypotension and tachycardia, as well as asymmetric abdominal distension. Laboratory investigations revealed a biological inflammatory syndrome and anemia. The abdominal CT scan showed significant aerated distension of the sigmoid colon. The sigmoid wall appeared to be dilated. Extra-digestive air bubbles were observed near the transition level, along with moderate intraperitoneal effusion. We found severe acute necrosis of the sigmoid colon and distal ileum, accompanied by a large volume of putrid ascitic fluid. The sigmoid was wrapped around the ilium. A Hartmann procedure was performed. The patient had an uneventful postoperative recovery.

**Discussion:**

ISK, also known as compound volvulus or double volvulus, is a rare and life-threatening intestinal obstruction caused by the twisting of the ileum and sigmoid colon around each other. The incidence of ISK is not known. The exact cause of ISK is unknown. Ingestion of heavy meals when the small intestine is empty. This may explain the high incidence during Ramadan. ISK is classified into four types according to the direction. This classification is more useful for predicting mortality and morbidity.

**Conclusions:**

ISK is a rare cause of intestinal obstruction and intestinal ischemia. It is important to differentiate between the Knott ileosigmoid and the simple volvulus of the sigmoid to avoid morbidity.

## Highlights

• Ileosigmoid knotting (ISK) is a rare and life-threatening intestinal obstruction caused by the twisting of the ileum and sigmoid colon around each other.
• The incidence of ISK is not known. It is more common in Africa, Asia, and the Middle East. The exact cause of ISK is unknown.
• Ingestion of heavy meals when the small intestine is empty. This may explain the high incidence during Ramadan. ISK is classified into four types based on the direction.

## 1 Introduction and importance

Ileosigmoid knotting (ISK) as a cause of acute intestinal obstruction is a very rare clinical entity [1]. Preoperative diagnosis is difficult, knowledge of its mechanism and the search for characteristic CT scan signs allows early diagnosis and therefore adapted care that reduces the potentially fatal consequences of this rare gastrointestinal emergency [2,3]. This case report, written according to SCARE guidelines [4], aims to highlight clinical presentation, diagnostic challenges, surgical intervention, and subsequent ISK management strategies during Ramadan fasting.

## 2 Case presentation

**Fig. 1:**
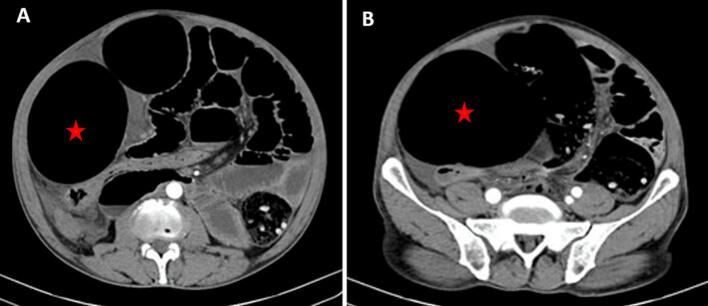
A - The abdominal CT scan revealed a whirl sign and significant distention of the haustral sigmoid loop, measuring 12 cm in diameter with parietal thinning. B - Sigmoid colonic loop with air-fluid level and beak appearance of the efferent limb of volvulus and dilated small bowel with poor contrast effect (Red star: dilated loop)

**Fig. 2:**
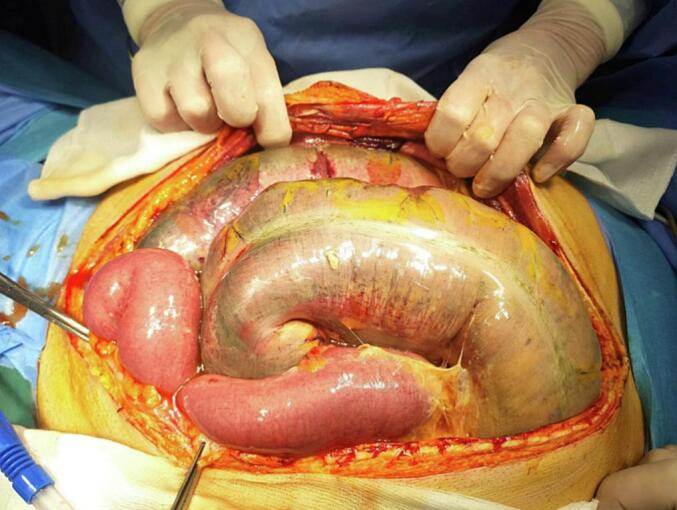
Intraoperative view showing digestive necrosis with gangrenous small intestine.

**Fig. 3:**
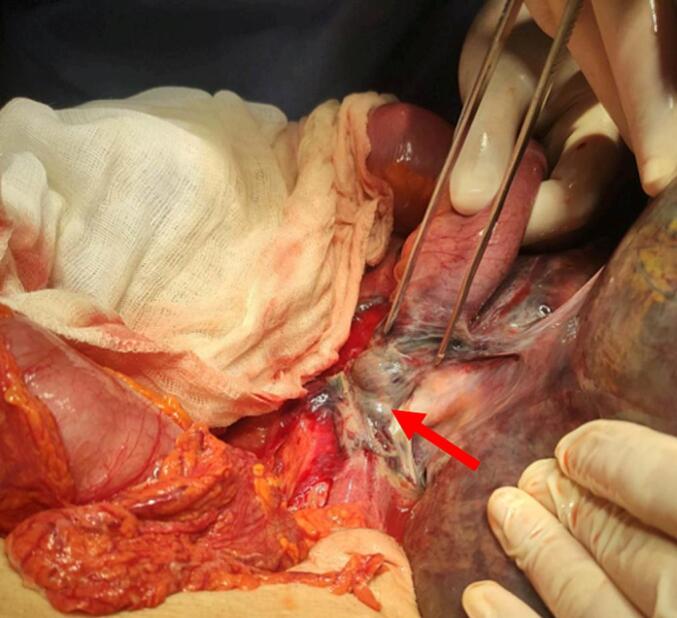
Intraoperative view of the ileal node (red arrow) around the base of the sigmoid, with necrosis of the twisted segments (8 cm of the small bowl and the sigmoid).

We report the case of a 52-year-old male who came to the emergency department during the Ramadan fast, with a three-day history of symptoms of intestinal obstruction. He complained of abdominal distention, vomiting, and absolute constipation. The patient indicated that he had no history of blunt trauma or previous surgeries. On physical examination, he had hypotension and tachycardia, as well as asymmetric abdominal distension, which is particularly pronounced in the left hypochondrium, we also observed the presence of diffuse tenderness, guarding, and rebound tenderness of the abdomen. The digital rectal examination reveals an empty rectum. Laboratory investigations revealed significant findings indicative of systemic inflammation and anemia. The white blood cell count was elevated at 12,000 cells/μL (reference range: 4000–10,000 cells/μL), consistent with leukocytosis. The level of C-reactive protein was markedly elevated at 150 mg/L (reference range: <5 mg/L), indicating an acute inflammatory response. Hemoglobin levels were reduced to 9.2 g/dL (reference range: 13.5–17.5 g/dL for men), which confirms anemia. Renal function tests, including creatinine and blood urea nitrogen, were within normal limits (creatinine: 0.7–1.2 mg/dL, BUN: 7–20 mg/dL), as well as liver function tests, suggesting no immediate organ dysfunction. The coagulation profiles were also within the expected range (PT: 11–13.5 s, INR: 0.8–1.2), ruling out coagulopathy. These findings raised suspicion of intestinal obstruction, prompting further diagnostic imaging and management. A chest CT scan of the abdomen showed significant aerated distention of the sigmoid colon, reaching a diameter of 12 cm (Fig. 1A), with a visible ‘whirl sign’ indicating a transition zone. The sigmoid wall appeared dilated, with adjacent twisted colonic segments showing a ‘bird's beak’ appearance, indicative of a volvulus (Fig. 1B). Extra-digestive air bubbles were observed near the transition level, along with moderate intraperitoneal effusion (Fig. 2). The patient underwent an emergency laparotomy. We found severe acute necrosis of the sigmoid colon and distal ileum, accompanied by a large volume of putrid ascitic fluid (Fig. 2). The sigmoid was wrapped around the ilium in a clockwise direction, thus creating a type II ileosigmoid node (Fig. 3). The sigmoid was excessively long and redundant, with a short mesenteric pedicle. The ileum extended to approximately 8 cm. A Hartmann procedure was performed, which involved the removal of the sigmoid colon and the creation of a colostomy. In addition, the necrotic ileum was excised, and a double barrel ileostomy was established. The patient had an uneventful postoperative recovery and was discharged from the hospital after 10 days. Plans were made for stoma reversal and colorectal anastomosis after ten weeks. During regular follow-ups, he remained symptom-free.

## 3 Discussion

Ileosigmoid knotting (ISK), also known as compound volvulus or double volvulus, is a rare and life-threatening intestinal obstruction caused by twisting of the ileum and sigmoid colon around each other [3]. The incidence of ISK is not known. It is more common in Africa, Asia, and the Middle East [2]. ISK occurs more frequently in men (80.2 %) than in women, with a peak incidence in middle age [4]. The exact cause of ISK is unknown, but several factors are believed to contribute, including the following.

• Anatomical abnormalities: Redundant sigmoid colon with narrow mesenteric pedicle, long mesentery of the small intestine, and hypermobile small intestine [5].
• Predisposing factors: Postoperative adhesions, Meckel's diverticulum with a band, ileocecal intussusception, trans mesenteric intestinal herniation, floating cecum, and malrotation [6].
• Dietary factors: Ingestion of heavy meals when the small intestine is empty. This may explain the high incidence during Ramadan [7]. However, our patient was a Muslim man who suffered from ISK during Ramadan. The relationship between Ramadan fasting and the development of ISK warrants further exploration, as dietary habits during this period –characterized by prolonged fasting followed by the ingestion of large meals –can influence gastrointestinal dynamics and predispose individuals to conditions such as volvulus. Fasting leads to extended periods of an empty small intestine, which, coupled with the consumption of heavy meals, could alter intestinal motility and increase susceptibility to knotting. This case underscores the need for increased clinical awareness during Ramadan, as fasting-related changes in intestinal physiology may contribute to rare but severe complications such as ISK. Understanding this association could guide preventive measures and early diagnostic strategies in fasting populations.
• Pregnancy: Late pregnancy has also been reported to be a predisposing risk factor for ISK due to intestinal displacement due to uterine enlargement. ISK is classified into four types according to the direction and involvement of the ileum and sigmoid colon [8]:
  • Type I: The ileum revolves around a passive sigmoid colon.
  • Type II: The colon revolves around the ileum (as in this case).
  • Type III: the iliac segment is part of the knot.
  • Undetermined: Unable to identify the active segment.

If the direction of rotation is known, an ‘a’ or ‘b’ suffix is added depending on whether it is clockwise or counterclockwise. This classification is mainly anatomical and does not influence the outcome [9]. Atamanalp proposed a new classification based on the age of the patient, the associated diseases, and the presence or absence of gangrene in the intestine [10]. This classification is more useful for predicting mortality and morbidity. Our case was classified as class IIa according to the anatomical classification and class IIIB according to the previous classification. Diagnosis Preoperative diagnosis of this life-threatening condition is difficult due to its rarity and confusing clinical and radiographic presentations; this is possible only in about 20 % of patients in the literature [3,5]. The patient typically presents with acute central abdominal pain; then the pain becomes constant and generalized, associated with vomiting. In 56 % of the cases, the patient presents hypovolemic shock [11,12]. Abdominal examination reveals moderate abdominal distention, tenderness, or palpation abdominal guards, with intestinal silence when intestinal necrosis is already established [12]. The plain X-ray can show multiple fluids from the small intestine on the left side and a dilated sigmoid colon on the right side. CT is the most reliable imaging modality for diagnosing ISK. The abdominal CT scan has a diagnostic precision of >92 %. It can show the characteristic “whirl sign” and twisted bowel segments [13]. The diagnosis of ileosigmoid knot is suggested by the presence of clinical and radiological findings of colonic obstruction and small bowel obstruction. It is essential to differentiate it from simple sigmoid volvulus which can lead to attempts to perform endoscopic decompression with a sigmoidoscope that can lead to perforation of the injury [12]. The mortality rate of the ileosigmoid knot in general may reach 48 %. Early diagnosis and prompt surgical intervention are crucial to managing ISK. Various surgical procedures are mentioned in the literature [14]. Anatomical and pathological intraoperative findings dictate the surgical procedure. The most common procedure involves resection of the necrotic bowel segments and restoration of intestinal continuity. The Hartmann procedure/colonostomy for the descending colon is indicated if both the ileum and the sigmoid are gangrenous [15,16]. The prognosis for ISK depends on the severity of the bowel necrosis and the timing of surgery. Early intervention improves the outcome and reduces mortality [17].

## 4 Conclusions

This case highlights the importance of recognizing ISK as a rare but severe cause of intestinal obstruction. It underscores the critical role of CT imaging in identifying key diagnostic features like the “whirl sign” and the need for timely surgical intervention. The Hartmann procedure, chosen based on the extent of bowel necrosis, emphasizes the importance of individualized surgical strategies. This case also raises awareness of potential fasting-related factors in ISK development, warranting further study.

## Patient consent

Written informed consent was obtained from the patient to publish this case report and accompanying images. On request, a copy of the written consent is available for review by the Editor-in-Chief of this journal.

## Provenance and peer review

Not commissioned, externally peer reviewed.

## Ethical approval

Ethical approval is exempt/waived at our institution.

## Guarantor

Mohamed Ali Chaouch.

## Research registration number

Not applicable.

## Funding

No funding.

## Author contribution

All the authors participated in the manuscript and validated the final version of the manuscript

## Conflict of interest statement

The authors declare no conflict of interest.
